# Advance lung cancer inflammation index (ALI) at diagnosis is a prognostic marker in patients with metastatic non-small cell lung cancer (NSCLC): a retrospective review

**DOI:** 10.1186/1471-2407-13-158

**Published:** 2013-03-27

**Authors:** Syed H Jafri, Runhua Shi, Glenn Mills

**Affiliations:** 1 Department of medicine, division of hematology/oncology, Louisiana State University Health, Shreveport, LA, USA; 2 Feist-Weiller Cancer Center, Shreveport, LA, USA

**Keywords:** Lung cancer, Inflammation, Neutrophil/lymphocyte ratio, Advanced lung cancer inflammation index

## Abstract

**Background:**

Systemic inflammation has been linked with cancer development, cancer cachexia and poor outcome. Advanced lung cancer inflammation index (ALI) was developed to assess degree of systemic inflammation at the time of diagnosis in metastatic non-small cell lung (NSCLC) cancer patients.

**Methods:**

In a single institution retrospective review 173 patients with metastatic NSCLC diagnosed between Jan 1 2000 and June 30 2011 were included. ALI was calculated as (BMI x Alb / NLR) where BMI = body mass index, Alb = serum albumin, NLR (neutrophil lymphocyte ratio, a marker of systemic inflammation). Patients were divided into low inflammation (ALI ≥ 18) and high inflammation (ALI < 18) groups. Kaplan-Meier method was used to estimate progression free survival and overall survival. Log-rank test were used to compare the survivals among various factors. Multivariate Cox regression was used to perform survival analysis in order to estimate the hazards ratio for various factors.

**Results:**

Among 173 patients median age was 57 years, 67% were male, 52% had adenocarcinoma. Patients with an ALI score of < 18 suggesting high systemic inflammation were significantly more likely to have more than 2 sites of metastatic disease, have poor performance status and less likely to receive any chemotherapy. Their median progression free survival and overall survival was 2.4 months and 3.4 months as opposed to 5.1 months and 8.3 months in patients with ALI >18 (P < 0.001). On multi-variate analysis ALI score of <18 (1.42, 95% CI 1.003-2.01) remained significantly associated with worse outcome.

**Conclusion:**

ALI (<18) at diagnosis is an independent marker of poor outcome in patients with advanced NSCLC.

## Background

Lung cancer is one of the most commonly diagnosed cancers in the United States. It is estimated that 226,160 new cases of lung cancer will be diagnosed and 160,340 will die from it in year 2012 alone [1]. 85% of lung cancer cases are classified as non-small cell lung cancer (NSCLC) and the rest (15%) are classified as small cell lung cancer(SCLC) [2]. NSCLC is further classified based on histology, 29% of diagnosed cases have squamous cell cancer (SCC) and approximately 32% have adenocarcinoma and the rest have other sub-types [3]. More than half of the patients (56%) with lung cancer at the time of diagnosis have advanced or metastatic disease [4] and even with chemotherapy have a median survival of one year or less [5].

Inflammation is recognized both as a condition that leads to cancer development and also as a condition that arises due to oncogenic changes in cancer cells [6]. The six hallmarks of cancer, distinctive and complimentary capabilities that enable tumor growth and metastatic dissemination are sustaining proliferative signaling, evading growth suppressors, resisting cell death, enabling replicative immortality, inducing angiogenesis and activating invasion and metastasis. Inflammation has been described as the underlying or enabling characteristic that promotes these hallmarks of cancer [7]. Systemic inflammation besides promoting tumor growth has also been shown to be responsible for many cancer related symptoms including cancer cachexia, anorexia, pain, debilitation and shortened survival [8].

There are various laboratory markers of systemic inflammation including plasma C-reactive protein concentration (CRP), hypoalbuminaemia and Glasgow Prognostic Score (GPS, which combines CRP and albumin), and absolute white cell and its components (neutrophils, neutrophils/lymphocyte ratio (NLR) and platelet/lymphocyte ratio (PLR) [9]. Of these, NLR have been shown to be a rapid and a simple parameter of systemic inflammation in critically ill intensive care unit (ICU) patients with a NLR > 5 suggesting ongoing systemic inflammation [10].

In patients with completely resected NSCLC, NLR have not only been shown to be associated with higher stage but also serve as an independent predictor of survival [11]. In another study of advanced colorectal cancer patients NLR of >5 was shown to be an independent predictor of overall survivals [9].

We wanted to evaluate if degree of systemic inflammation at the time of diagnosis in patients with advanced NSCLC can be a prognostic marker for outcome. For this purpose we developed a simple index based on patient’s height, weight, serum albumin and NLR from the time of diagnosis. We call this advanced lung cancer inflammation index (ALI). The purpose of this study was to see if advanced lung cancer inflammation index (ALI) at the time of diagnosis can predict survival outcomes in patients with newly diagnosed metastatic NSCLC.

## Methods

All patients diagnosed with stage IV NSCLC at our institution between Jan 1 2000 and Jun 30 2011 were screened for this retrospective review. Patients with a prior history of non-small cell lung cancer presenting with relapse, prior history of other cancers in preceding 5 years and those with incomplete medical information or follow up were excluded. Height, weight, absolute neutrophil count, absolute lymphocyte count and serum albumin obtained from the medical records were recorded from the date of diagnosis or from the date closest to the date of diagnosis. Most data points were from within 2 weeks of the date of diagnosis and none were more than 6 weeks after diagnosis.

Radiological response and date of progression were taken from the medical records as judged at that time by the treating physician. Date of death was obtained from tumor registry and or from medical records. Progression free survival was defined as the time period between date of diagnosis till radiological progression or deterioration in performance status rendering patient ineligible for further treatment or death. Overall survival was defined as the time period between date of diagnosis and the date of death or date of last contact if exact date of death is unavailable. The study was conducted after obtaining approval from Louisiana state university-Shreveport institutional review board.

### Statistical methods

Clinical indicators were calculated as follows. Student t test was used to compare means and Pearson Chi-square test or the Fisher’s exact test was used to compare proportion where appropriate. Kaplan-Meier method was used to estimate progression free survival and median overall survival. Log-rank test were used to compare the survivals among various factors. Multivariate Cox regression was used to perform survival analysis in order to estimate the hazards ratio for various factors.

### Advanced lung cancer inflammation index (ALI)

The Area under a receiver operating characteristic (ROC) curve was used to identify the factors that could predict patient’s response to the treatment or not. Ideally a survival event should be used in this ROC analysis, however 97.8% of patients are dead and only 4 patients are alive, therefore responses to treatment or not was used in the ROC analysis. The areas under ROC curves, c statistics, were 0.67, 0.61, 0.65, 0.70 and 0.74 for absolute neutrophil count, absolute lymphocyte count, serum albumin, BMI, and ALI respectively. Therefore based on the highest value of area under ROC curve, ALI was used to predict responses or not to the treatment in the ROC analysis. The single optimal cut point for ALI score is 18.4 in which the sensitivity and specificity is 77.3% and 63.9%. This optimal point was selected by using the minimum distance between the point 100% sensitive and 100% specific and any point on the ROC curve. Advance lung cancer inflammation index (ALI) was defined as follows:

$$\begin{array}{l} \mathrm{Advance}\;\mathrm{lung}\;\mathrm{cancer}\;\mathrm{inflammation}\;\mathrm{index}\\ \;\left(\mathrm{ALI}\right)=\left(\mathrm{BMI}\right)\times \left(\mathrm{Alb}\right)/\mathrm{NLR} \end{array}$$

Where:

$$\mathrm{Body}\;\mathrm{mass}\;\mathrm{index}\;\left(\mathrm{BMI}\right)=\mathrm{weight}\;\left(\mathrm{lb}\right)/{\left[\mathrm{height}\;\left(\mathrm{in}\right)\right]}^{2}\times 703$$

Alb = serum albumin in g/dLNeutrophil lymphocyte ratios (NLR) = ANC/ALCANC: Absolute neutrophil countALC: Absolute lymphocyte count.

In this study, for simplicity the ALI score were dichotomized as < =18 and >18 in the respectively analysis. All p-value < 0.05 were considered statistically significant. The SAS system 9.3 (SAS institute Inc. Gary, NC) was used to performed all the analyses.

## Results

A total of 173 patient records with complete medical information and follow up were included in the final analysis. Patient’s characteristics are shown in Table 1. Median age was 57 years old with (range from 34 to 88 years). Two thirds of patients were male. African Americans constituted approximately half of our patient population which is consistent with demographics in the city.

**Table 1:** Patient characteristics with advanced NSCLC

	**N = 173 (%)**
**Age (median, range)**	57 (34–88)
**SEX**	
Male	116 (67)
Female	57 (33)
**RACE**	
African American	90 (52)
White	83 (48)
**Performance status (PS)**	
0-1	130 (76)
2-4	42 (24)
**Histology**	
Adenocarcinoma	91 (52)
Non-adenocarcinoma	82 (47)
**Number of metastatic sites**	
1-2	93 (54)
>2	80 (46)
**Chemotherapy**	
No chemo	59 (34)
Any chemo	114 (66)
**First response assessment**	
Response to chemotherapy	41 (23)
Stable disease	24 (14)
Progression	39 (23)
Decline in performance status	69 (40)
**Survival (months)**	
Median progression free survival	3.7
Median overall survival	5.1

NSCLC: Non-small cell lung cancer.

The most common histology was adenocarcinoma (52%), followed by squamous cell (20%), poorly differentiated carcinoma (10%) and various other histologies as shown in Table 2. Epidermal growth factor receptor (EGFR) mutation status was not available for most (75%) patients with adenocarcinoma as our cohort included patients from as early as 2000. Of those patients that had EGFR mutation information available it was found to be negative in 65%, positive in 9% and 26% of samples had insufficient material to test EGFR status (Table 3).

**Table 2:** NSCLC distribution by histology

	**N = 173 (%)**
**Adenocarcinoma**	91 (52)
**Squamous cell**	34 (20)
**Poorly differentiated**	17 (10)
**Adeno-squamous**	11 (6)
**Non-small cell cancer**	6 (3)
**Large cell carcinoma**	5 (3)
**Carcinoma**	5 (3)
**Bronchoalveolar**	2 (1)
**Squamous cell-Small cell**	1 (0.5)
**Mucoepidermoid**	1 (0.5)

*NSCLC*: Non-small cell lung cancer.

**Table 3:** EGFR mutation status of adenocarcinoma patients

	**N = 91 (%)**
**Not available**	**68 (75)**
**Tested**	**23 (25)**
EGFR negative	15 (65)
Insufficient material	6 (26)
EGFR positive	2 (9)

EGFR: Epidermal growth factor receptor.

Different sites of metastatic disease included lung, liver, brain, bones, adrenal glands, spleen and retroperitoneal lymph nodes and each was considered a separate metastatic site. (54%) of patients had up to 2 metastatic sites and the rest had more than 2. At the time of diagnosis most patients (76%) had an ECOG performance status of 0–1 and 24% had PS 2 or higher.

About a third (35%) of all patients could not receive any chemotherapy due to poor PS or co-morbid illnesses. Median number of chemotherapy cycles was 2 with a range of 0–24 cycles. At first response assessment 23% had response to chemotherapy, 14% had stable disease and 23% had progression of disease and 40% had decline in PS making them ineligible for further chemotherapy. Median progression free survival for the entire group was 3.7 months and overall survival was 5.1 months.

In order to get an estimate of ongoing systemic inflammation at the time of diagnosis we calculated ALI for each patient using the formula described above. Range of ALI was 0.46-158.4.Patients were then divided around 50th percentile mark into two groups, those with ALI of < 18 (more inflammation) and those with ALI of ≥ 18 (less inflammation). Their characteristics are listed in Table 4. There was no significant difference in age at diagnosis, sex and race and tumor histology between the two groups.

**Table 4:** Patient characteristics and outcome between those with advanced lung cancer inflammation index ALI score <18 (more inflammation) and those with ALI score > 18 (less inflammation) at the time of diagnosis

**Variable**	**ALI < 18 (n = 83) (%)**	**ALI ≥ 18 (n = 90) (%)**	**P = value**
**Age (median)**	56	58	0.26 (a)
**Male**	57 (69)	59 (65)	0.74 (b)
**AA**	42 (51)	48 (53)	0.76 (b)
**Adenocarcinoma**	51 (61)	53 (59)	0.75 (b)
**PS 2-4**	**27 (32)**	**15 (17)**	**0.02 (b)**
**No chemotherapy**	**41 (49)**	**18 (20)**	**<0.001 (b)**
**>2 metastatic sites**	**48 (58)**	**32 (35)**	**0.003 (b)**
**Response to chemotherapy**	**15 (18%)**	**50 (56%)**	**<0.0001(b)**
**Median PFS**	**2.4**	**5.1**	**<0.001 (c)**
**Median OS**	**3.4**	**8.3**	**<0.001 (c)**

(a) Student t-test, (b) Chi-square test, (c) Log rant test, *AA* = African American, *PS* = Performance status, *PFS* = Progression free survival, *OS* = Overall survival. Significant results shown in bold.

**Figure 1:**
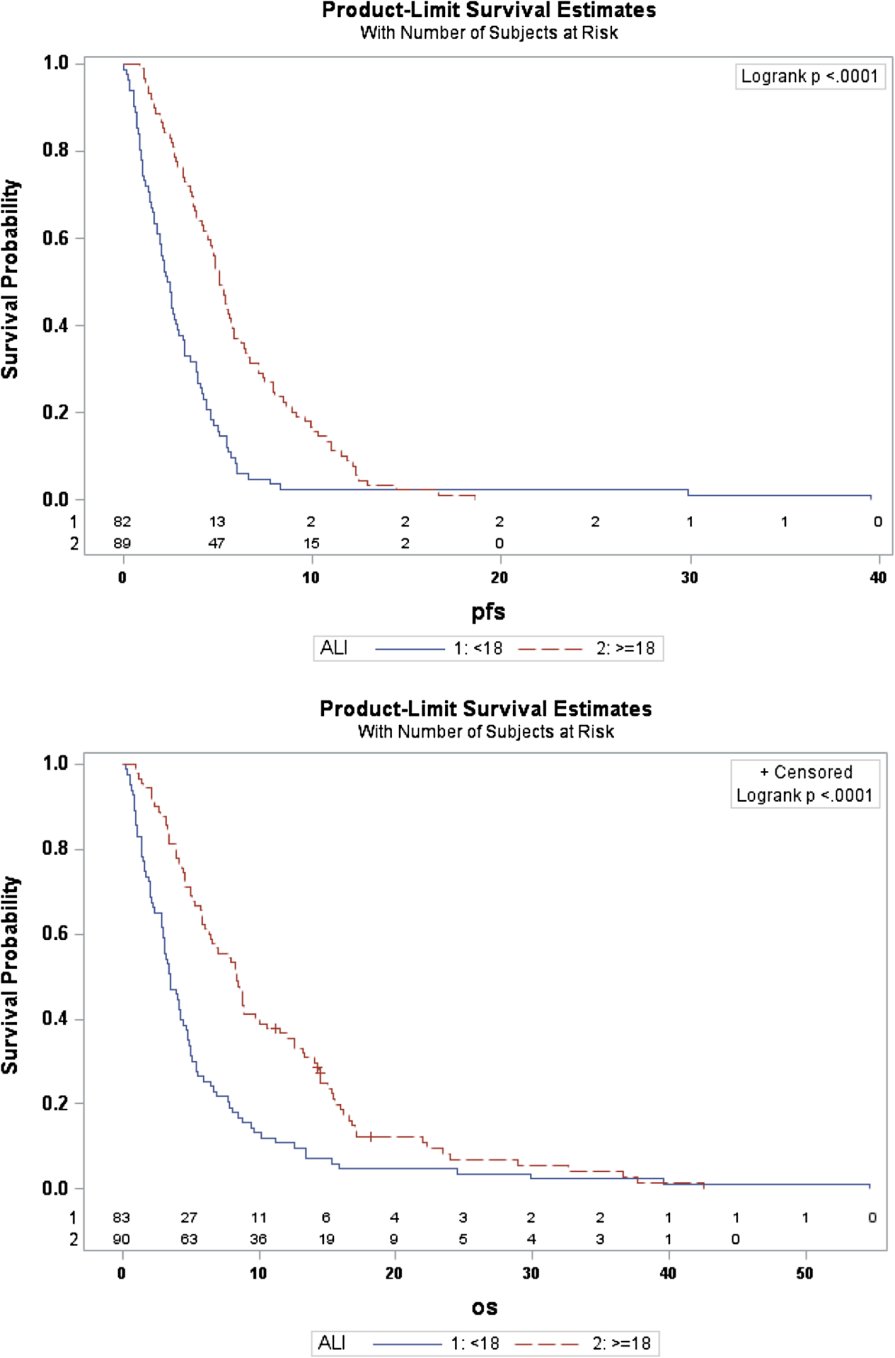
**Kaplan-Meir survival curve for (PFS) progression free survival and (OS) overall survival between patients with advanced lung cancer inflammation index of (< 18, high inflammation) and (>18, low inflammation).** (Time in months).

Compared to patients with ALI score of ≥ 18 patients with an ALI score of < 18 were significantly more likely to have more than 2 sites of metastatic disease at the time of diagnosis (P = 0.003), have poor PS (P = 0.02), less likely to receive any chemotherapy (P <0.001) and very low response to chemotherapy (P < 0.0001). They also did worse in terms of PFS and OS. Patients with ALI of <18 had a median PFS of 2.4 months and OS of 3.4 months. Patients with ALI of > 18 had a median PFS of 5.1 months and OS of 8.3 month. Both PFS and OS were highly significant between two groups (P < 0.001) (Figure 1).

In univariate analysis of overall survival age < 60 years (HR = 1.42, 95% CI 1.03-1.95, P = 0.03), Absolute lymphocyte count (ALC) < 1 (HR = 1.82 (1.16-2.87, P = 0.009), ALI of < 18(HR = 1.97, 95% CI 1.45-2.6, <0.0001) and no chemotherapy (HR =2.36, 95% CI 1.7-3.2, <0.0001) were associated with significantly worse outcome. Similarly PS of 0–1 (HR = 0.64, 95% CI 0.44-0.93, P = 0.01) and 1–2 metastatic sites (HR = 0.54, 95% CI 0.39-0.73, P = 0.0001) were associated with better outcome (Table 5).

**Table 5:** Univariate analysis of clinical characteristics on progression free and overall survival in patients with metastatic non-small cell lung cancer

**Variable**	**PFS: HR, (95% CI)**	**P = value**	**OS: HR, (95% CI)**	**P = value (a)**
**Age < 60**	**1.31 (0.96-1.79)**	0.08	1.42 (1.03-1.95)	0.03
**Gender (Female/Male)**	1.14 (0.82-1.58)	0.41	0.97 (0.7-1.35)	0.88
**Race (AA/White)**	0.89 (0.65-1.20)	0.44	1.08 (0.8-1.47)	0.59
**Histology (Adeno/Other)**	1.03 (0.76-1.4)	0.82	1.01 (0.74-1.38)	0.90
**PS (0-1/ 2–4)**	**0.62 (0.43-0.89)**	**0.009**	**0.64 (0.44-0.93)**	**0.01**
**Mets (1-2/ >2)**	**0.47 ( 0.34-0.64)**	**<0.0001**	**0.54 (0.39-0.73)**	**0.0001**
**No chemotherapy**	**2.25 (1.62-3.13)**	**<0.0001**	**2.36 (1.7-3.2)**	**<0.0001**
**ALC <1**	**1.61 (1.02-2.53)**	**0.03**	**1.82 (1.16-2.87)**	**0.009**
**NLR < 5**	**0.58 (0.42-0.8)**	**0.001**	**0.57 (0.41-0.79)**	**0.0008**
**ALI < 18**	**2.26 (1.65-3.09)**	**<0.0001**	**1.97 (1.45-2.68)**	**<0.0001**

a) Cox-proportional hazard regression model.

*Abbreviations*: *AA* African Americans, *Adeno* Adenoacrinoma, *ALC* Absolute lymphocyte count, *ALI* Advanced lung cancer inflammation index, *PS* performance status, *Mets* metastatic sites, *NLR* Neutrophil/lymphocyte ratio. Significant results shown in bold.

On multi-variate cox regression analysis adjusted for sex, race, PS and histology ALI is a significant predictor of both PFS and OS. ALI score of < 18 has a hazard ratio of 1.66 (1.18-2.33, P = 0.003) for PFS and 1.42 (95% CI 1.003-2.01, P =0.047) for OS. With an ALI < 18 at the time of diagnosis, the risk of dying increases by 42% as compared to ALI ≥ 18 (Table 6).

**Table 6:** Multi-variate analysis of clinical characteristics on progression free and overall survival in patients with metastatic non-small cell lung cancer

**Variable**	**PFS: HR (95% CI)**	**P = value**	**OS : (95% CI)**	**P = value (a)**
**Age <60 years**	**1.46 (1.03-2.07)**	**0.03**	**1.64 (1.14-2.36)**	**0.006**
**No chemotherapy**	**2.04 (1.38-3.01)**	**0.0003**	**2.12 (1.42-3.18)**	**0.0002**
**Mets 1-2**	**0.56 (0.39-0.79)**	**0.001**	**0.69 (0.49-0.97)**	**0.03**
**ALI <18**	**1.66 (1.18-2.33)**	**0.003**	**1.42 (1.003-2.01)**	**0.047**

a) Cox proportional hazard regression model, Variables adjusted included sex, race, Performance status, histology.

*ALI* advanced lung cancer inflammation index, *Mets* metastatic sites. Significant results shown in bold.

## Discussion

Cancer and inflammation are closely linked and many inflammatory conditions increase the risk of cancer development like inflammatory bowel disease and increased risk of colorectal cancer, hemochromatosis and liver cancer [12] and sjogren’s syndrome and lymphoma [13]. Similarly in a nested case control study of 592 lung cancer patients and 670 controls pre-diagnostic elevated C-reactive protein was found to be associated with increased risk of lung cancer development (odds ratio [OR], 1.98; 95% CI, 1.35 to 2.89; *P*-trend < .001 for fourth quartile [Q4, ≥5.6 mg/L] *v* Q1 [< 1.0 mg/L]) [14].

Systemic inflammation is also linked to poor outcome in cancer patients. In 102 unresctable pancreatic cancer patients elevated C-reactive protein at the time of diagnosis was found to be an independent poor prognostic factor. Median survival of those with an acute phase protein response (CRP > 10 mg/L, n = 45) was 66 days compared with 222 days for those with no acute phase protein response (n = 57, P = 0.001, Mann–Whitney U test) [15].

In patients with inoperable stage III and IV NSCLC, an inflammatory score called Glasgow prognostic score (based on serum CRP and albumin) was shown to be of prognostic value in predicting outcome (HR 1.70,95% CI 1.23-2.35, P = 0.001) [16]. In an another study of mostly stage III and few stage IV NSCLC patients elevated CRP at the time of diagnosis was associated with increased weight loss (P = 0.004), reduced albumin (P = 0.001), reduced performance status (p = 0.06), increased fatigue (P = 0.01) and reduced survival (HR 1.93,95% CI 1.41-2.6, P <0.001) [17].

Most patients with lung cancer at the time of diagnosis have metastatic disease. ALI was developed to assess ongoing systemic inflammation in these patients. Both weight loss and a low serum albumin are associated with ongoing systemic inflammation [17]. NLR, a marker of systemic inflammation has been found to predict poor outcome in patients with different cancers including lung cancer [11]. ALI was developed using these markers into an easy to calculate unified index called advanced lung cancer inflammation index. Moreover as opposed to Glasgow prognostic score it is exclusively derived from patients with stage IV or metastatic lung cancer [16].

There was no difference in ALI score based on tumor histology suggesting that both adenocarcinoma and non-adenocarcinoma generate similar degree of systemic inflammation. In our study patients with ALI score of < 18 suggesting high systemic inflammation were found to have more sites of metastatic disease, have a poor performance status , receive no chemotherapy and had an overall poor PFS (2.4 vs 5.1 months, P < 0.001) and OS (3.4 vs 8.3 months < 0.001). This data suggests progression of cancer through the body leads to higher level of systemic inflammation resulting in poor performance status, less chance of getting chemotherapy and ultimately earlier demise.

In our cohort a third of all patients never received any chemotherapy including 50% of patients with ALI <18. Many such patients have poor performance status at the time of diagnosis hence excluding them from standard treatment as well as participation in clinical trials. As our data suggests many such patients have high degree of systemic inflammation and it may be the reason for their poor PS and less change of receiving any chemotherapy. In light of this data gauging systemic inflammation in patients with advanced NSCLC at the time of diagnosis could be an important clinical consideration.

In multi-variate analysis age < 60 years, more than two metastatic sites, no chemotherapy and ALI < 18 were independently associated with worse outcome. Of these age and number of metastatic sites are non-modifiable variables. High systemic inflammation as represented by ALI <18 hence represents a potential therapeutic area for improving patient outcome.

In our cohort patients with age < 60 years had worse outcome. The reason for this is unclear to us. They were also more likely to have > 2 sites of metastatic disease (54% vs 32%, P = 0.004). There was no difference in ALI score between those with age < 60 or more than 60. One explanation could be that in this cohort, those who got diagnosed with cancer at a young age may have a more biologically aggressive disease, hence more metastatic sites and worse outcome.

If systemic inflammation is a poor prognostic marker as suggested by GPS and our study, then combining anti-inflammatory agents with chemotherapy makes sense. In a randomized placebo controlled phase III study of advanced NSCLC patients platinum doublet chemotherapy was combined with celecoxib 400 mg twice daily or placebo. However there was no difference in median progression free survival (HR 0.8, 95% CI, 0.6-1.1; P = 0.25) or median overall survival (HR 0.9; 95% CI, 0.6-1.2; P = 0.32) between the two groups. Moreover cyclooxygenase-2 (COX-2) expression was neither a prognostic nor a predictive marker of use of celecoxib [18]. In this study most patients had PS of 0–1 suggesting low systemic inflammation. Perhaps combining anti-inflammatory agents with chemotherapy will be more beneficial in patients with high level of systemic inflammation.

There are several drawbacks of using ALI. This data is based on retrospective chart review hence many data points may not be accurately recorded. Patients height, weight, albumin, ANC and ALC were not necessarily from the same date though most were within two weeks of diagnosis. Also ALI is a continuous variable hence there may be little difference between ALI of 17 and 19 though they fall on the side of high risk and low risk respectively based on current study.

This study sheds light on the importance of assessing or gauging systemic inflammation in advanced NSCLC patients as a prognostic marker. However ALI should be validated in a prospective cohort study. In such study ALI should be compared with tumor specific mutations like EGFR, KRAS, EML4-ALK etc. to see which mutations are associated with higher systemic inflammation.

As far as treatment is concerned patients with higher systemic inflammation at the time of diagnosis should be considered high risk and should be started on systemic chemotherapy without delay perhaps initially with single agent only, provided their overall performance status and organ function allows such treatment to be given. Further studies should be conducted combining anti-inflammatory agents with chemotherapy in patients with high degree of systemic inflammation to see if this combination is beneficial.

## Conclusion

Advanced lung cancer patients should have an assessment of degree of systemic inflammation at the time of initial clinical evaluation. Advanced lung cancer inflammation index (ALI) is an easy to calculate tool to assess ongoing systemic inflammation in these patients. High systemic inflammation as judged by ALI of < 18 is an independent prognostic marker of poor outcome. It also correlates with widespread metastatic disease, poor performance status and less chance of receiving any chemotherapy. However, it should be validated in a prospective cohort study.

## Abbreviations

Alb: Serum albumin; ALI: Advance lung cancer inflammation index; ALC: Absolute lymphocyte count; ANC: Absolute neutrophil count; BMI: Body mass index; CI: Confidence interval; CRP: C-reactive protein; ECOG: Eastern cooperative oncology group; EGFR: Epidermal growth factor receptor; EML4-ALK: Echinoderm microtubule-associated protein-like 4-Anaplastic lymphoma kinase; GPS: Glasgow prognostic score; HR: Hazard ratio; ICU: Intensive care unit; NLR: Nuetrophil/lymphocyte ratio; NSCLC: Non-small cell lung cancer; PFS: Progression free survival; PS: Performance status; PLR: Platelet/lymphocyte ratio; OR: Odds ratio; OS: Overall survival; SAS: Statistical analysis system; SCC: Squamous cell cancer; SCLC: Small cell lung cancer.

## Competing interests

All authors declare that they have no competing interests.

## Authors’ contributions

SHJ designed the study, developed ALI, collected data, helped in statistical analysis, wrote the manuscript. RS performed statistical analysis and wrote the manuscript. GM reviewed the data and contributed to the manuscript. All authors read and approved the final manuscript.

## Pre-publication history

The pre-publication history for this paper can be accessed here:

http://www.biomedcentral.com/1471-2407/13/158/prepub
